# Solitary skin metastasis from sarcomatoid carcinoma of the bladder: a case report

**DOI:** 10.1186/1752-1947-5-484

**Published:** 2011-09-28

**Authors:** Antonio Manzelli, Silvia Quaresima, Piero Rossi, Athanasios Petrou, Edoardo Ricciardi, Nicholas Brennan, Michael Kontos, Giuseppe Petrella

**Affiliations:** 1 Dipartimento di Chirurgia, Cattedra di Chirurgia Generale Università Tor Vergata, Direttore Prof. Giuseppe Petrella, Policlinico Tor Vergata, Viale Oxford 81, 00133 Roma, Italia

## Abstract

**Introduction:**

Cutaneous metastases from carcinomas of the bladder are very rare. They are related to advanced stages of the disease and have poor prognosis with low survival rates. The common treatment modality of cutaneous metastases from a primary bladder cancer is wide local excision followed by chemotherapy.

**Case presentation:**

We report a case of solitary skin metastasis from a rare type of urinary bladder carcinoma in a 68 year-old Caucasian man. Urinary bladder carcinoma metastasizing to the skin is an uncommon finding despite the high incidence of this tumor. Skin metastasis generally presents in the late stages of this disease and indicates a poor outcome.

**Conclusions:**

Because of the extremely aggressive malignant potential of sarcomatoid carcinomas, the indications for a transurethral resection of the bladder should be carefully assessed and suitable therapeutic strategies should be examined further.

## Introduction

The incidence of cutaneous metastasis from primary urinary malignances is reported from 1.1% to 2.5%. The most common are from kidney cancer (3.4-4%) followed by urinary bladder cancer (0.84-3.6%) and prostate cancer (0.36-0.7%) [1]. Usual sites of metastasis of urinary malignancies include lung, bone, liver and regional nodes [2]. Very few cases of skin metastasizing from urinary bladder are reported in the literature. This type of localization is rare, generally presenting in the late stages of disease and indicates a poor outcome. We report one case of cutaneous metastasis from sarcomatoid carcinoma of urinary bladder, a very rare histological type, with metastatic localization to the thoracic wall.

## Case Presentation

**Figure 1:**
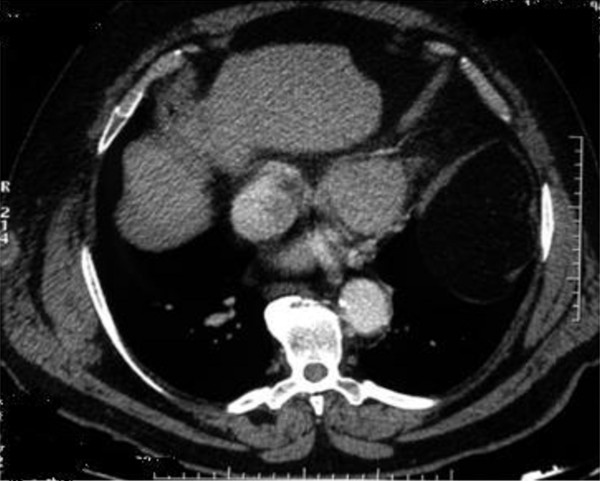
**CT scan showing a contrast enhanced hypodermic 38 × 22 mm nodular lesion on the right chest**.

**Figure 2:**
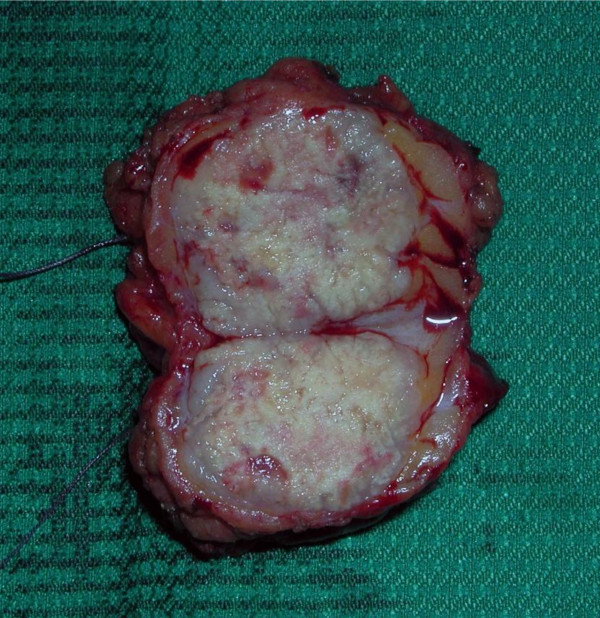
**Surgical specimens of chest wall nodule**.

**Figure 3:**
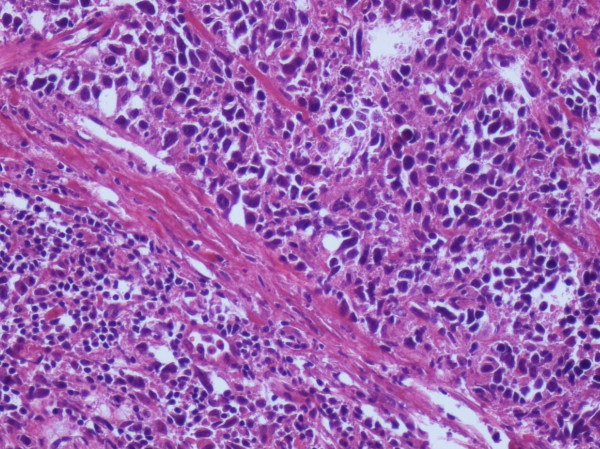
**Sarcomatoid invasion of the bladder muscularis/EE × 20**.

**Figure 4:**
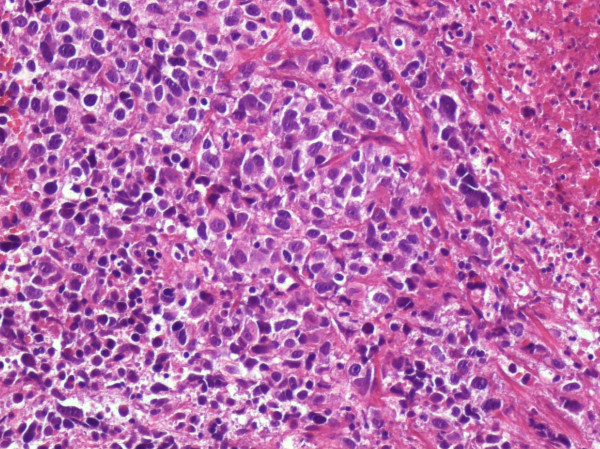
**Bladder round neoplastic elements/EE × 20**.

**Figure 5:**
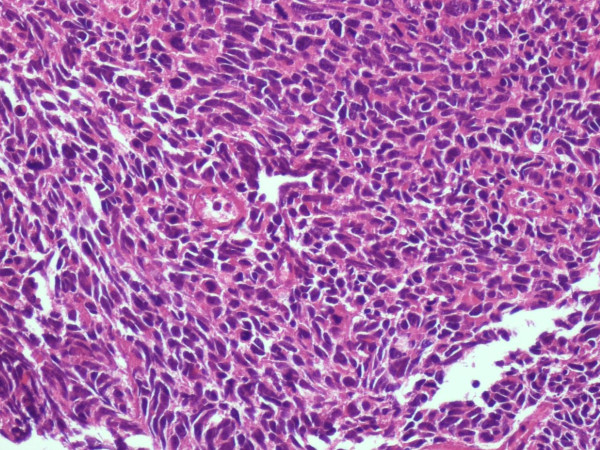
**Bladder sarcomatoid aspect/EE × 20**.

**Figure 6:**
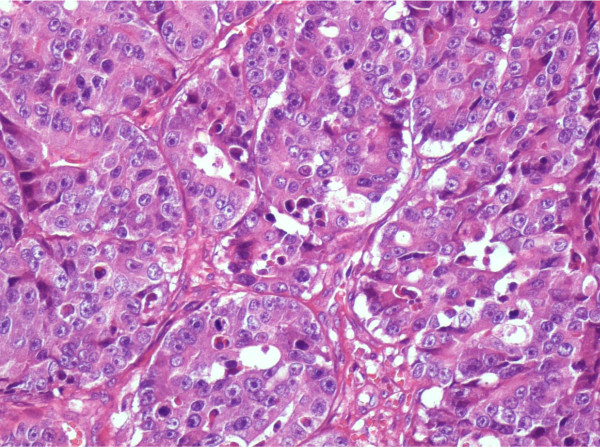
**Metastatic skin lesion aspect/EE × 20**.

A 68 year-old Caucasian man was admitted in our department complaining of gross haematuria. A cystoscopic examination found a 2.5 cm solid lesion located on the posterior wall of the bladder. A total body Computed Tomography (CT) scan was performed and showed a bladder lesion with loco-regional node enlargement. The CT scan revealed a hypodermic 38 × 22 mm nodular lesion located on the right chest wall with increased enhanced contrast (Figure 1). Cytological characterization of this lesion was obtained with a fine needle aspiration biopsy (FNAB) and "epithelial type cells with nuclear atypia" were found. Considering the CT scan results and the cytology report, a transurethral resection of the bladder (TURB) lesion was performed, along with surgical resection of the chest wall nodule (Figure 2). The histological diagnosis of the surgical specimen revealed sarcomatoid carcinoma invading the bladder musculature staged pT3aN3M1 and graded G3 (Figure 3, Figure 4, Figure 5). The skin lesion specimen showed poorly differentiated neoplastic infiltration with morphologic aspects of urothelial tissue with immunochemistry positivity for CK7 and cerb-B2 and immunochemistry negativity for CK20 CD117 and TTF-1. The TURB specimen showed neoplastic elements which were poorly differentiated, round and spindle shaped and with a high mitotic index (70 mitosis/10 HPF) (Figure 6). Small segments of these elements demonstrated epithelial type immunochemistry (CK7 and CK20 positive) whilst the major part of the neoplasm was composed of sarcomatoid type differentiated cells positive for desmin and negative for cytokeratins. The immunochemistry was also cromogranine A, smooth muscle actin, CD3, CD20, CD117, EGFR negative. The proliferative index evaluated with Ki67 was positive in the 60-70% of the sarcomatoid cells and the Cerb-B2 was positive at cytoplasmic membrane staining of the epithelial component and was negative in the sarcomatoid component. The histopathological report was summarized as an invasive poorly differentiated bladder carcinoma metastasis with a component of mixed, giant and spindle, sarcomatoid cells. Once recovered from surgeryg the patient received four cycles of chemotherapy consisting of gemcytabine, carboplatin and paclitaxel (Taxol). At six months post-surgical follow-up, a repeat CT scan showed, despite these treatments, a progression of loco-regional nodal disease and pulmonary metastasization.

## Discussion

Cutaneous metastases are generally associated with carcinomas invading the bladder musculature(T3a) or to a local advanced neoplasm (T3b/T4), although the literature reports a few cases of cutaneous metastasis in early stage bladder cancer [3]. Presence of cutaneous localization from urinary bladder cancer is highly correlated to large metastatic disease [4]. Prognosis after cutaneous metastasis appear generally poor with a median survival of 13 to 14 monthsfor patients treated by chemotherapy, although there is one sporadic case in the literature reporting survival at 34 months [5,6]. Wide surgical excision, as a curative and diagnostic attempt, is considered the first line procedure in these patients. In the treatment of metastatic bladder cancer, single agent chemotherapy using methotrexate, doxorubicin, vinblastine or cisplatin produce response rates in 15 to 25% of patients, whilst multiple agent chemotherapy treatment increases this to 50 to 70% of cases[7]. The combination of gemcytabin, paclitaxel and cisplatin produce response rates in 78% of cases and a complete remission in 28% of the patients producing a median survival rate of 24 months [8]. Alternative combinations of adjuvant therapies are reported in the literature. Craig et al reports a successful case with complete clinical resolution of two metastatic skin lesions in a patient submitted to a cystoprostatectomy for bladder carcinoma, using local irradiation [8]. Kubata et al also discuss a case of complete resolution in a patient treated with bleomycin electrochemotherapy. [9]. Although we need to consider that a non-operative clinical plan in these patients leads to certain disease progression, a single case in the literature describes a case of cutaneous metastasis with spontaneous regression [10]. However, this unusual subtype of cancer still remains a rare histological carcinoma variant where pathological diagnosis is often very difficult with a complex and extensive immunohistochemistry and genetic pattern as described by Terada in his recent publications [11,12].

The most prominent clinical characteristic of a sarcomatoid carcinoma of the urinary bladder is the extreme aggressive behavior. However, if the stage and the patient's clinical condition indicate surgery as appropriate, then the therapy of choice will be a radical surgical therapy. When surgery is not an option, palliation with radiotherapy is indicated. Further studies are necessary before we can make a conclusion on the therapeutic strategies for sarcomatoid carcinomas of the bladder.

## Conclusion

In conclusion, sarcomatoid carcinoma of the urinary bladder is a rare malignancy with a poor clinical prognosis. At the present time, it seems appropriate to treat in the same manner as conventional high-grade transitional cell carcinoma (TCC) of the bladder with similar degrees of invasion. In this group of patients it is important to recognize the possibility of metastasis at uncommon sites. This condition is highly correlated with an advanced oncological staging or with an aggressive histopatological grading of disease and indicates a very poor outcome for the patient.

## Consent

Written informed consent was obtained from the patient for publication of this case report and accompanying images. A copy of the written consent is available for review by the Editor-in-Chief of this journal.

## Competing interests

The authors declare that they have no competing interests.

## Authors' contributions

AM, SQ, PR, ER, MK and GP were the surgical and pathological team involved in the case. AP and NB wrote and edited the manuscript. All authors read and approved the final manuscript.
